# Not All Embolizations Are Created Equally in the Management of Posterior Epistaxis: Discussion of Safety Measures Avoiding Neurological Complications

**DOI:** 10.1155/2020/5710313

**Published:** 2020-08-20

**Authors:** Mareike Franke, Jasper Franke, Christian Saager, Sven Barthel, Randolf Riemann, Kersten Mueckner

**Affiliations:** ^1^Diagnostic and Interventional Radiology, Dr. Hancken Clinic, 21680 Stade, Germany; ^2^Department of Radiology, Wesling Hospital Minden, University Hospital of the Ruhr University Bochum, 32429 Minden, Germany; ^3^53 N Studios, 21682 Stade, Germany; ^4^Otorhinolaryngology, Head and Neck Surgery, Elbe Hospitals, 21680 Stade, Germany

## Abstract

Today, there are still no uniform guidelines for the treatment of epistaxis. Furthermore, it is widely debated whether embolization or surgical approaches should be the first choice of treatment for intractable posterior epistaxis after conservative measures have failed. In several meta-analyses, it is reported that endoscopic sphenopalatine artery ligation and embolization have similar success rates, but embolization was associated with more severe neurological complications. Regarding existing literature, there are many comparative analyses of surgical methods but none for embolization protocols. Against this backdrop of a lack of uniform standards in embolization techniques, we present a retrospective evaluation of what has emerged to be best procedural practice for endovascular treatment of epistaxis in our department using microsphere particles and microcoils, in particular regarding precaution measures to avoid neurological complications. In our retrospective data analysis of 141 procedures in 123 patients, performed between 2008 and 2019, we find success rates very similar to those reported in other studies (95.1% immediate-stop-of-bleeding success and 90.2% overall embolization success) but did not encounter any major neurological complication opposed to other reports. We suggest some aspects of our protocol as precaution measure to avoid neurological complications. More generally and perhaps even more importantly, we make a strong case for standardization for embolization techniques to the level of details in surgical procedure standardization to enable an apples to apples comparison of embolization techniques to each other and of intervention vs. surgery.

## 1. Introduction

Epistaxis is a common medical condition but is rarely a direct cause for hospital admission. Up to 60% of adult population experience nasal bleeding at some stage of their lives, but only 6% need medical help [1].

In most cases, the bleeding starts from the anterior septal area (Little area), which is vascularized by Kiesselbach's plexus. As the Little area is readily accessible, hemorrhage from this region can usually be managed by applying pressure to the nostrils, chemical or electrocautery, topical hemostatic or vasoconstricting agents, cryotherapy, or anterior nasal packing. However, severe, intractable bleeding usually arises from a posterior source, causing conservative measures to fail [2].

**Figure 1:**
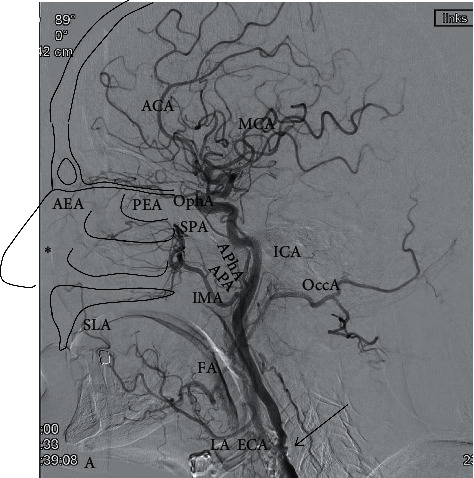
Arterial supply of nasal cavity. Digital subtraction angiography via the common carotid artery shows branches of the external and internal carotid artery (ECA and ICA; 90° LAO). For better understanding of the anatomy some anatomical structures like the nasal conchae, the floor of nasal cavity, nose, and frontobasis are drawn schematically. The major part of blood supply for the nasal cavity is provided via branches of the ECA. Especially the sphenopalatine artery (SPA), and end-branch of the internal maxillary artery (IMA), is the main blood supply for the nasal cavity and for Kiesselbach's plexus (asterisk). The roof of the nasal cavity is supplied by the anterior ethmoidal artery (AEA) and posterior ethmoidal artery (PEA), which are branches of the ophthalmic artery (OphA), i.e., branches of the internal carotid artery (ICA). The blood supply to the floor of the nasal cavity originates from ascending palatine arteries (APA) from the facial artery (FA) and descending palatine artery (DPA) from IMA. Little supply to Kiesselbach's plexus comes from the superior labial artery (SLA), an end-branch of the FA. And, finally minor supply to the posterior area of the nasal cavity is provided by the ascending pharyngeal artery (APhA), which originates from the ECA. Note the calcification of proximal ICA (arrow). Also shown are occipital artery (OccA), ophthalmic artery (OPhA), and intracranial arteries like anterior and middle cerebral artery (ACA and MCA). As ICA and ECA both contribute to the blood supply of the nasal cavity sometimes, there are some “dangerous” anastomoses, which can cause blindness or stroke when accidentally embolized during the procedure. These potentially “dangerous” anastomoses include the artery of the foramen rotundum, the middle meningeal artery, the accessory meningeal artery, the ethmoidal arteries, the APhA, and of course the communications between the FA and OPhA[3].

In general, for sufficient therapeutic management of anterior or posterior epistaxis, a profound anatomical knowledge of the branches of the external carotid artery and arterial blood supply of the nasal cavity is absolutely necessary (Figure 1).

An option to control such posterior bleeding is the application of anterior and posterior packs (AP packs). These packs have been reported to have a success rate between 48% and 83% [4–6]. As they can lead to nasal trauma, vagal response, aspiration, infection, allergic reactions, and airway obstruction they should be applied with care and under patient monitoring [7, 8].

If conservative measures fail, reducing the blood supply to the sinonasal area is an option. This can be achieved either with a surgical or endovascular approach. Different surgical methods have been described: there is a wide range from transantral artery ligation, submoucous resection, external carotid artery (ECA) ligation, and anterior ethmoidal artery ligation to endoscopic sphenopalatine artery ligation [9]. So far, endoscopic sphenopalatine artery ligation has the most favorable adverse effect profile and success rate compared with other surgical approaches [9–13].

Endovascular treatment of epistaxis was first presented in 1974 as an alternative to surgery by Sokoloff et al. [14] and consisted of particle embolization of the ipsilateral internal maxillary artery (IMA). Later, Lasjaunias et al. [15] refined the technique stressing the need for a standardized angiographic and therapeutic protocol. Due to dangerous anastomoses between the external and internal carotid arteries with the risk of embolic material entering the internal carotid artery or ophthalmic artery, the procedure comes with the risk of severe neurologic complication, such as hemiplegia, ophthalmoplegia, facial paralysis/paresthesia, or blindness [16, 17]. Cases of facial necrosis are published as well [18–20].

As with surgery, there are many different protocols and techniques for embolization described (reviewed in [9] and [21]). Despite this multitude of techniques and protocols, no comparing study exists. The following methods have been individually described in literature [19, 21, 22]:

- Embolization of the ipsilateral IMA and sphenopalatine artery (SPA) or
- Embolization the ipsi- and contralateral IMA/SP or
- Embolization of the ipsilateral or both ipsi- and contralateral IMA/SPA in combination with embolization of the ipsilateral or both ipsi- and contralateral facial artery.

There are also many different embolization materials used (polyvinyl alcohol, coils, microspheres, gelfoam, etc. [21]). In general, the success rates of endovascular therapy have been reported between 75 and 92% [9, 21].

Today, the possible treatment options for intractable epistaxis are widely debated. While endovascular treatment is arguably easier on the patient requiring no general anesthesia and has a shorter hospital stay [23], the presence of “dangerous anastomoses” and the associated risk of cerebrovascular accident (CVA) is feared [3, 16].

We believe the main contribution of this paper to be two-fold: (1) we retrospectively evaluated 141 mostly microsphere-based procedures performed on 123 patients at our hospital, thus contributing to a still very thin report base. (2) We find that—besides an encouragingly high success rate and a tolerable rate of rebleedings—not a single CVA occurred in our sample. We believe this to be due to the precautionary measures developed as part of our interventional protocol using calibrated microspheres and coils.

## 2. Materials and Methods

Between 2008 and 2019, 141 percutaneous endovascular embolization procedures for epistaxis were performed on 123 patients (80 male, 43 female, mean age 66, ranging from 18 to 90 years). Patients were referred to us by the hospital's otorhinolaryngologist after failed conventional treatment, involving anterior and posterior nasal packing and/or cauterization. Preinterventional imaging was not mandatorily performed. Patients suffered from severe epistaxis (as classified in [24]). Approximately 20% of patients required blood transfusion due to chronic blood loss over a time period of several days with a drop in hemoglobin levels. Interventions were performed with AP packs in situ. None of the patients which underwent intervention were in hemorrhagic shock.

All interventions were performed on an emergency basis in the angiographic facility of our department (until 2008 using a Siemens Axiom Artis angiography unit; from 2008 until today using a Philips Allura XP angiography unit). Patients received a mild sedation (1–2.5 mg Midazolam i.v.). Monitoring of blood pressure and pulse oximetry was performed during the procedure. Patient assessment was performed according to Table 1.

**Figure 2:**
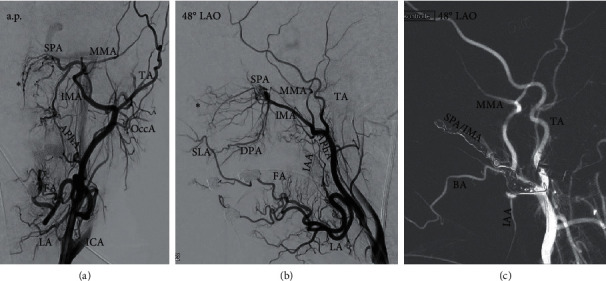
Embolization of the left internal maxillary artery/sphenopalatine artery (IMA/SPA) in a 49-year-old female patient with hereditary hemorrhagic telangiectasia. A.p. (a) and 48° LAO (b, c) Angiogram of left external carotid artery (ECA) shows arterial supply of nasal cavity via IMA. (a, b) Main supply of Kiesselbach's plexus (asterisk) is provided via SPA, but there also collaterals to the superior labial artery (SLA, a branch of the facial artery FA). (c) Angiogram after embolization with micospheres and coils: there is no contrast flow in the SPA. Particle embolization of the SPA/distal IMA needs to be done very carefully to avoid accidental nontarget embolization of the middle meningeal artery (MMA) with possible hazardous anastomoses to ICA branches. Also shown is the labial artery (LA), temporal artery (TA), buccal artery (BA), occipital artery (OccA), descending palatine artery (DPA), and inferior alveolar artery (IAA).

Pathologic coagulation parameters had to be balanced by the referring department (INR < 1.5, platelets > 60,000 per *μ*l). The embolization procedure was performed according to Table 2 (Figure 2).

**Figure 3:**
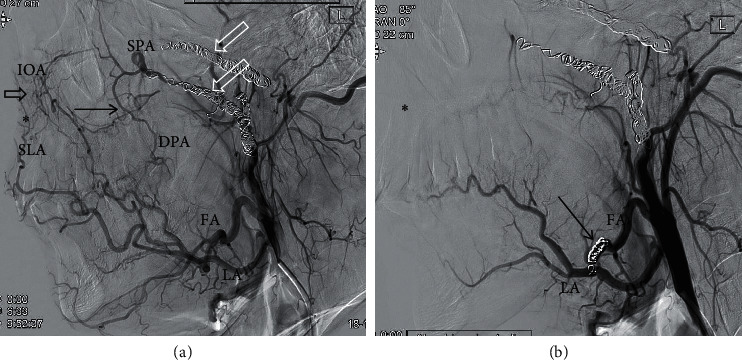
Embolization of left facial artery (FA) in a 25-year-old male patient with epistaxis and hereditary hemorrhagic telangiectasia having had a double-sided embolization 5 years ago. (a) Angiogram of the external carotid artery (ECA) shows coils in both SPA/IMA (right and left; white open arrows) after a successful double-sided embolization in 2014. Examination shows an extended collateral network via the facial artery (FA) in 2019. The collateral network consists of cross-connections of the superior labial artery (SLA)/infraorbital artery (IOA, open black arrow) and branches of FA/descending palatine artery (DPA, black arrow). (b) Coil embolization of FA was performed (black arrow). Control angiogram after embolization shows no significant contrast flow in the collateral network and Kiesselbach's plexus. Also shown is the labial artery (LA).

**Figure 4:**
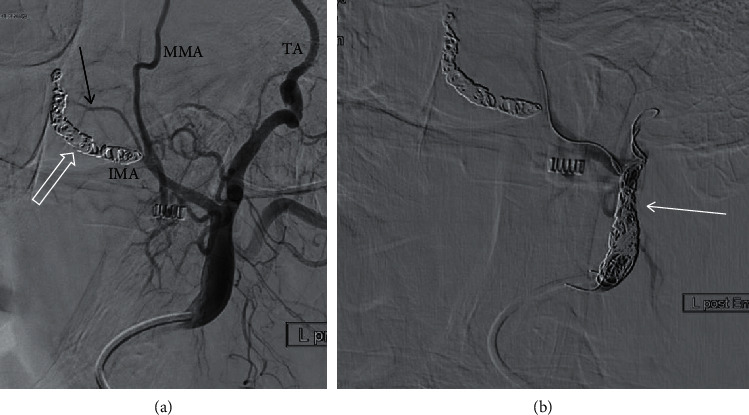
Re-embolization of left external carotid artery (ECA) due to an accessory artery from IMA in a 72-year-old male patient with epistaxis (risk factor: anticoagulation). (a) Angiogram of ECA shows coils in the internal maxillary artery (IMA) and sphenopalatine artery (SPA, open white arrow) after embolization 53 days earlier. However, there is an accessory artery rising from of the proximal IMA (black arrow), then running to the dorsal part of the nasal cavity, potentially being responsible for the rebleeding. (b) Decision was made to embolize the ECA to stop the blood supply to this artery. No particles were used. Embolization was performed with coils (white arrow). Also shown are middle meningeal artery (MMA) and temporal artery (TA).

For synergistic effects, AP packs were left overnight and removed the next day. 6 Patients had an embolization only with coils, without particles due to re-embolization of contralateral side during the first five days or an embolization of other arteries than SPA/distal IMA (Figures 3 and 4).

Data were analyzed in terms of etiologies of epistaxis, duration of procedure (i.e., duration of vulnerable time with catheters in the carotid arteries), overall embolization success, immediate-stop-of-bleeding success, and complications. Overall embolization success of endovascular treatment was defined as successful one- or double-sided embolization of IMA/SPA avoiding surgery during a follow-up period of at least 6 months. Note that this definition of success also includes cases of patients which had to be re-embolized as the first embolization did in some cases not stop the bleeding immediately, but in all cases of embolization success, the patient avoided the more invasive treatment of surgery. Immediate-stop-of-bleeding success was classified as stopping the bleeding immediately for the next five days. While in some cases, the patients had to be re-embolized or required subsequent surgery; we considered it a success because the patient had at least five days during which they did not lose blood and did not require blood transfusion and could recover without AP packs. These two types of success are individually important but represent two distinct dimensions of benefit to the patient and are thus evaluated separately. Adverse effects were classified in major and minor complications according to [25].

## 3. Results and Discussion

### 3.1. Results

As described in the literature, many cases of epistaxis were idiopathic (Table 3).

Due to noncompliance, 4 patients needed general anesthesia. In terms of overall embolization success, one-sided or double-sided embolization safely controlled hemorrhage in 111 of 123 patients, which needed no further surgery (Table 4). Technical success rate was 99.3% with one technical failure. In this case, a catheterization of the IMA was not possible. Immediate-stop-of-bleeding success could be achieved in 117 of 123 patients (95.1%). Early rebleeding within the first five days after first embolization could be observed in six patients (4.8%). In 16 cases, rebleeding occurred more than five days after embolization (13.0%).

No life-threatening adverse event occurred (Table 4). Especially no CVA could be observed. The average procedure duration (i.e., duration of vulnerable period with catheters in carotid arteries) was 17.4 minutes, ranging from 4 to 31 minutes.

### 3.2. Discussion

There are some meta-analyses stating that there are more neurological complications for endovascular treatment of epistaxis compared to surgery [3] (in contrary to other [26]) and case series/reports showing neurological and facial complications of endovascular treatment [16, 18]. This leads to the opinion that endoscopic ligation of the sphenopalatine artery should be the method of choice and embolization should be performed in cases where surgical treatment fails or the patient has a high anesthetic risk. In our center, however, the endovascular treatment after failure of conservative measure is routine, which emerged also from the discussion with our otorhinolaryngologists. It has been investigated that there is a high risk of contamination of medical staff with patient`s blood during surgery [27, 28]. Since we are frequently asked to treat epistaxis, we developed a protocol with high safety standards to avoid CVA. All patients were hemodynamically stable with nasal packs in situ. The INR/platelet count had to be normalized before therapy (INR < 1.5 and platelets >60,000 *μ*l). Despite being admitted with diagnosis of intractable epistaxis, all patients received a dose of 5,000 I.E. heparin at the procedure's beginning to avoid clot formation in catheters or adherent to the wires [29]. As complication rate increases with arteriosclerotic vessel changes and procedure time [30–32], we try to keep the time of carotid catheterization as low as possible. Therefore, we prefer a “no-touch of ICA” strategy: In contrary to other publications (e.g., [15, 33]), we do not perform a selective angiogram of the ICA because of calcifications in the passing region with the risk of thrombus release and in addition increasing time of procedure. Only an angiogram of the carotid bifurcation is performed in every patient to identify ICA stenosis or occlusion which could lead to recruitment of ECA/ICA collateral pathways requiring special attention. To detect the dangerous ICA/ECA-anastomoses, we perform an accurate angiogram of ECA and of the pterygopalatinal segment of IMA.

Another safety measure is continuous pressure flushing of the guiding catheter in the ECA to prevent blood clots. Additionally, we carefully rinse the microcatheter after application of particles before coil embolization of SPA and IMA to avoid dislocation of particles. In general, you have to keep in mind that stained micorparticles may be visible through the skin if injected into superficial arteries [34]. Therefore, we make sure to use nonstained particles for head and neck embolizations. The size of microspheres for embolization is carefully chosen. While smaller particles are able to penetrate more distally into capillary beds, they can also cause injury to the vasa nervorum resulting in cranial nerve palsies or enter the intracranial circulation through ECA/ICA anastomoses. When using microspheres, it is recommended to oversize these particles for head and neck embolization compared with polyvinyl alcohol (PVA) particles [20]. We use 500 *μ*m calibrated microparticles for embolization. The application should be performed in highest magnification and dose of continuous fluoroscopy to detect a reflux instantly to prevent nontarget embolization. Particle embolization is halted when flow starts to slow. If there was any doubt about (1) ICA/ECA-anastomoses, (2) the need for a second embolization during the first week, or (3) the need to embolize other arteries than IMA/SPA, no particles were used at all to avoid CVA or facial necrosis. The embolization with coils only had a slightly higher failure rate than particle plus coil-embolization (one of 6 patients needed further surgery; success rate: 83.3%). Some reports criticize the use of coils in general, because the possibility of repetition of distal embolization could be lost [23]. We use particle and coil embolization of SPA/IMA as we have the impression that, by doing so, we reduce also the residual flow and pressure via the IMA in prevention of a relapse. Furthermore, revascularization after embolization often occurs either via the contralateral side or via the facial artery, thus these arteries can be easily embolized during a second procedure. Should a revascularization occur via the ethmoidal arteries, a surgical therapy would be required.

Rebleeding is a known problem for the treatment of epistaxis. Primary double-sided or triple-artery embolizations are described in the literature postulating that the rate of early recurrences decreases with the number of arteries embolized [19]. However, this group had a certain rate of nose necrosis and several cases of facial edema. A case analysis from 2018 even found out that bilateral particle embolization including facial artery was the treatment method associated with a significant risk of complications [35]. With the exception of patients with hereditary hemorrhagic telangiectasia, one-sided embolization was very efficient in most cases. All 3 patients with hereditary hemorrhagic telangiectasia needed at least a double-sided embolization, and two of them had further surgery after primary bleeding control. In general, these patients are difficult to treat.

The only observed major complications were access site complications with a slightly increased rate of arterial occlusions (1.4% vs. 0.1–0.9% [36]). The reason for this could be the introduction of vascular closure devices in 2008 with no extensive experience during the first month of use. A few cases of headache after embolization could be well handled with analgesics.

The limitation of our case series is of course the small case number of 141 procedures. Although this is to our knowledge one of the largest primary case series in literature, it does not allow postulating general rules at statistical significance. Our protocol has been proved to be safe and effective with a primary efficiency of 95% with no neurological complications. So, we would like to present our protocol or aspects of our protocol as a suggestion for the reader or as basis of a further discussion.

## 4. Conclusions

Despite reports in the literature describing a higher rate of cerebrovascular complications compared to surgery, we think that when implementing the abovementioned security precautions, embolization of sphenopalatine artery with microcoils and microparticles is a safe, fast, and effective method to stop posterior epistaxis without requiring general anesthesia. Today, there is still no uniform standard in embolization of epistaxis and a complex data situation with different techniques and materials described. We feel the strong need for a systematic comparison of different embolization techniques and recommend further investigations to compare outcome/complication rate of different protocols to develop a uniform standard.

## Figures and Tables

**Table 1:** Patient assessment before and after intervention.

Indication	Refractory posterior epistaxis after 48 h conservative treatment, confirming indication by otorhinolaryngologist and radiologist
Laboratory assessment	Creatinine, thyroid-stimulating hormone, platelet count, hemoglobin, INR
Sedation/anesthesia	Mild sedation with midazolam or general anesthesia when necessary (e.g., restless patient)
Assessment in the operating suite	The patient placed lying down, infusion (500 ml sodium chloride), head sedated with no radiopaque materials in beam path (e.g., remove dental prosthesis) One operator, one operator assistant, and an additional suite technician and anesthesiologist when necessary
Assessment after procedure	AngioSeal/ExoSeal occlusion of vessel access (common femoral artery), manual compression for 10–15 minutes, compression bandage overnight, ultrasound control of groin the next day

**Table 2:** Standard protocol of procedure (see also Figure 2).

Step 1	Femoral artery access, 5 F introducer sheath
Step 2	Administration of 5000 I.E. heparin to avoid blood clots
Step 3	Catheterize common carotid artery (CCA) with 5 F guide catheter (100 cm) and hydrophilic guide wire
Step 4	Angiogram of the carotid bifurcation (40° RAO or LAO, resp.)
Step 5	Catheterize external carotid artery (ECA) with guide wire and guide wire—tip of catheter approx. 2 cm above the bifurcation
Step 6	Angiogram of ECA to find hazardous anastomoses or other unusual causes of epistaxis, e.g., AVM, tumor, pseudoaneurysm of sinonasal arteries
Step 7	Pressure flushing of guiding catheter in ECA with heparinised normal saline and introduce microcatheter and microwire
Step 8	Identify internal maxillary artery (IMA) and sphenopalatine artery (SPA) and catheterize
Step 9	Angiogram of IMA/SPA (“dangerous anastomoses” and MMA (Figure 2)
Step 10	When there are no hazardous anastomoses, embolize SPA/pterygopalatine segment of IMA with calibrated microparticles in dilution with contrast medium (500 *μ*m) until flow begins to slow. Important: avoid reflux, especially avoid embolization of MMA (headaches and hazardous anastomoses; Figure 2) When there are ECA-ICA anastomoses, directly go to step 12
Step 11	Rinse the microcatheter properly to avoid dislocation of microparticles
Step 12	Embolize SPA and distal IMA with microcoils
Step 13	Control angiogram
Step 14	Quick removal of all catheters from the carotid arteries
Step 15	Removal of introducer sheath, AngioSeal, or ExoSeal occlusion of vessel, compression bandage until the next day
Step 16	Nasal packing is left intact overnight and removed for inspection for bleeding the next day

**Table 3:** Etiologies of epistaxis.

Etiology	No. of patients
Idiopathic epistaxis (no risk factor could be detected)	45
Anticoagulant therapy	26
Hypertension	23
Anticoagulant therapy and hypertension	8
Low platelet count	1
Alcohol withdrawal	1
Surgical complication/preliminary surgery	7
Malignant tumor	8
Trauma	1
Hereditary hemorrhagic telangiectasia	3

**Table 4:** Outcome of patients in a follow-up period of at least 6 months.

*In 111 patients*, *embolization controlled active hemorrhage and no surgery was necessary*	90.2%
(i) In 101 patients, a one-sided embolization was sufficient to stop the bleeding	
(ii) 2 patients had a rebleeding **at the third and fourth days** after intervention and needed embolization of contralateral side; embolization of the contralateral site was performed without particles to avoid facial necrosis, since there was only a short interval to the first embolization	
(iii) 4 patients had a rebleeding **at the 7**^**th**^**till 14**^**th**^**day** after intervention and needed embolization of the contralateral side	
(iv) 2 patients had a rebleeding **after 31 and 32 days** after intervention and needed embolization of contralateral side	
(v) 1 patient had a rebleeding **after 5 months** after intervention and needed embolization of the contralateral side	
(vi) 1 patient with hereditary hemorrhagic telangiectasia had a successful double-sided embolization in 2014 (rebleeding after 36 days) and a rebleeding in 2019; in 2019, an embolization of the facial artery was performed without particles (Figure 3)	

*12 patients needed additional surgery*	9.7%
(i) 4 patients had **surgery at the following** day after one-sided embolization due to recurrence of bleeding	
(ii) 1 patient had a rebleeding **7 days** after one-sided embolization and received surgery	
(iii) 3 patients had a rebleeding after **7, 22, and 28 days**, respectively, after successful endovascular treatment, and contralateral embolization failed to stop the rebleeding, surgery was performed	
(iv) 1 patient had a rebleeding **one week** after successful embolization; embolization failed due to lack of possibility to catheterize contralateral IMA (technical failure); the patient received surgery	
(v) 2 patients with hereditary hemorrhagic telangiectasia had a rebleeding **49 and 97 days**, respectively, after a successful embolization; contralateral embolization was not successful in stopping the bleeding; surgery was performed	
(vi) 1 patient had a successful embolization of left IMA/SPA and **53 days** later had a rebleeding on the left side; an embolization of the left external carotid artery (ECA) was performed due to an atypical accessory artery (Figure 4); **7 days** later, a rebleeding occurred and embolization of the right IMA/SPA was performed; due to recurrence of bleeding, surgery was performed	

## Data Availability

All data on which the conclusion of this study are based (procedural notes, patient count, preexisting conditions, success rate, complication rate, rebleeding rate, and failure rate from retrospective analysis of anonymized data) are included within the article.
